# Complete genome sequence of the haloalkaliphilic, obligately chemolithoautotrophic thiosulfate and sulfide-oxidizing γ-proteobacterium *Thioalkalimicrobium cyclicum* type strain ALM 1 (DSM 14477^T^)

**DOI:** 10.1186/s40793-016-0162-x

**Published:** 2016-06-03

**Authors:** Ulrike Kappler, Karen Davenport, Scott Beatson, Alla Lapidus, Chongle Pan, Cliff Han, Maria del Carmen Montero-Calasanz, Miriam Land, Loren Hauser, Manfred Rohde, Markus Göker, Natalia Ivanova, Tanja Woyke, Hans-Peter Klenk, Nikos C. Kyrpides

**Affiliations:** School of Chemistry and Molecular Biosciences, The University of Queensland, Brisbane, Australia; Los Alamos National Laboratory, Bioscience Division, Los Alamos, New Mexico, USA; Centre for Algorithmic Biotechnology, St. Petersburg State University, St. Petersburg, Russia; DOE Joint Genome Institute, Walnut Creek, California, USA; Oak Ridge National Laboratory, Oak Ridge, Tennessee, USA; School of Biology, Newcastle University, Newcastle upon Tyne, UK; Central Facility for Microscopy, HZI – Helmholtz Centre for Infection Research, Braunschweig, Germany; Leibniz Institute DSMZ – German Collection of Microorganisms and Cell Cultures, Braunschweig, Germany; Department of Biological Sciences, Faculty of Science, King Abdulaziz University, Jeddah, Saudi Arabia

**Keywords:** Aerobic, Obligate chemolithoautotroph, Sulfur oxidizer, Gram-negative, Mono Lake, *Piscirickettsiaceae*, CSP 2008

## Abstract

*Thioalkalimicrobium cyclicum* Sorokin *et al*. 2002 is a member of the family *Piscirickettsiaceae* in the order *Thiotrichales*. The γ-proteobacterium belongs to the colourless sulfur-oxidizing bacteria isolated from saline soda lakes with stable alkaline pH, such as Lake Mono (California) and Soap Lake (Washington State). Strain ALM 1^T^ is characterized by its adaptation to life in the oxic/anoxic interface towards the less saline aerobic waters (mixolimnion) of the stable stratified alkaline salt lakes. Strain ALM 1^T^ is the first representative of the genus *Thioalkalimicrobium* whose genome sequence has been deciphered and the fourth genome sequence of a type strain of the *Piscirickettsiaceae* to be published. The 1,932,455 bp long chromosome with its 1,684 protein-coding and 50 RNA genes was sequenced as part of the DOE Joint Genome Institute Community Sequencing Program (CSP) 2008.

## Introduction

Strain ALM 1^T^ (= DSM 14477 = JCM 11371) is the type strain of the species *Thioalkalimicrobium cyclicum* [1], one of four species in the genus *Thioalkalimicrobium* [2]. The most prominent feature of *T. cyclicum* is its ability to live chemolithoautotrophically in the aerobic surface waters of a mixolimnion lake. Cultures of strain ALM^T^ were first isolated from Mono Lake water samples taken from the sulfide-oxygen interface layer at a depth of 19 – 25 m [1]. The species epithet for the organism was derived from the Latin adjective *cyc.li’cum*, cyclus, pertaining to the circle-like shape of the cells. For a short time after the initial description of the organism it was known as “*Thialkalimicrobium cyclicum*” until the Judical Commission of the International Committee on Systematics of Prokaryotes restored the correct genus name at the X^th^ International IUMS Congress of Bacteriology and Applied Microbiology in Paris (France) [3]. Here we present a summary classification and a set of features for *T. cyclicum* ALM 1^T^ (DSM 14477^T^), together with the description of the genomic sequencing and annotation of the genome. Sequencing was done within the DOE JGI CSP 2008 for analysis of three type strains of alkaliphilic sulfur oxidizers.

## Organism information

### Classification and features

A representative genomic 16S rDNA sequence of *T. cyclicum* ALM1^T^ was compared using NCBI BLAST [4] under default settings (e.g., considering only the HSPs from the best 250 hits) with the most recent release of the Greengenes database [5] and the relative frequencies of taxa and keywords (reduced to their stem [6]) were determined, weighted by BLAST scores. The most frequently occurring genera were *Thiomicrospira* (74.7), *Thioalkalimicrobium* (11.2), *‘**Thialkalimicrobium**’* (8.4), *Hydrogenovibrio* (3.8) and *‘Thiovibrio’* (1.9 %) (49 hits in total). Regarding the single hit to sequences from members of the species, the average identity within HSPs was 98.7 %, whereas the average coverage by HSPs was 96.4 %. Regarding the single hit to sequences from other members of the genus, the average identity within HSPs was 98.5 %, whereas the average coverage by HSPs was 92.6 %. Among all other species, the one yielding the highest score was *‘**Thialkalimicrobium**sibericum’* (AF126549), which corresponded to an identity of 98.6 and an HSP coverage of 96.3 %. (Note that the Greengenes database uses the INSDC (= EMBL/NCBI/DDBJ) annotation, which is not an authoritative source for nomenclature or classification, inverted commas indicate species names that are not approved.) The highest-scoring environmental sequence was DQ900619 (Greengenes short name ‘Sulfur-oxidizing Soap Lake (Washington State) meromictic haloalkaline unprecedented sulfide content lake water isolate ASL1ASL1 str. ASL1’, where ‘meromictic’ denotes a lake with separate, oxic and anoxic waterzones that do not intermix), which showed an identity of 99.7 % and an HSP coverage of 89.3 %. Environmental samples which yielded hits of a higher score than the highest scoring species were not found.

**Fig. 1:**
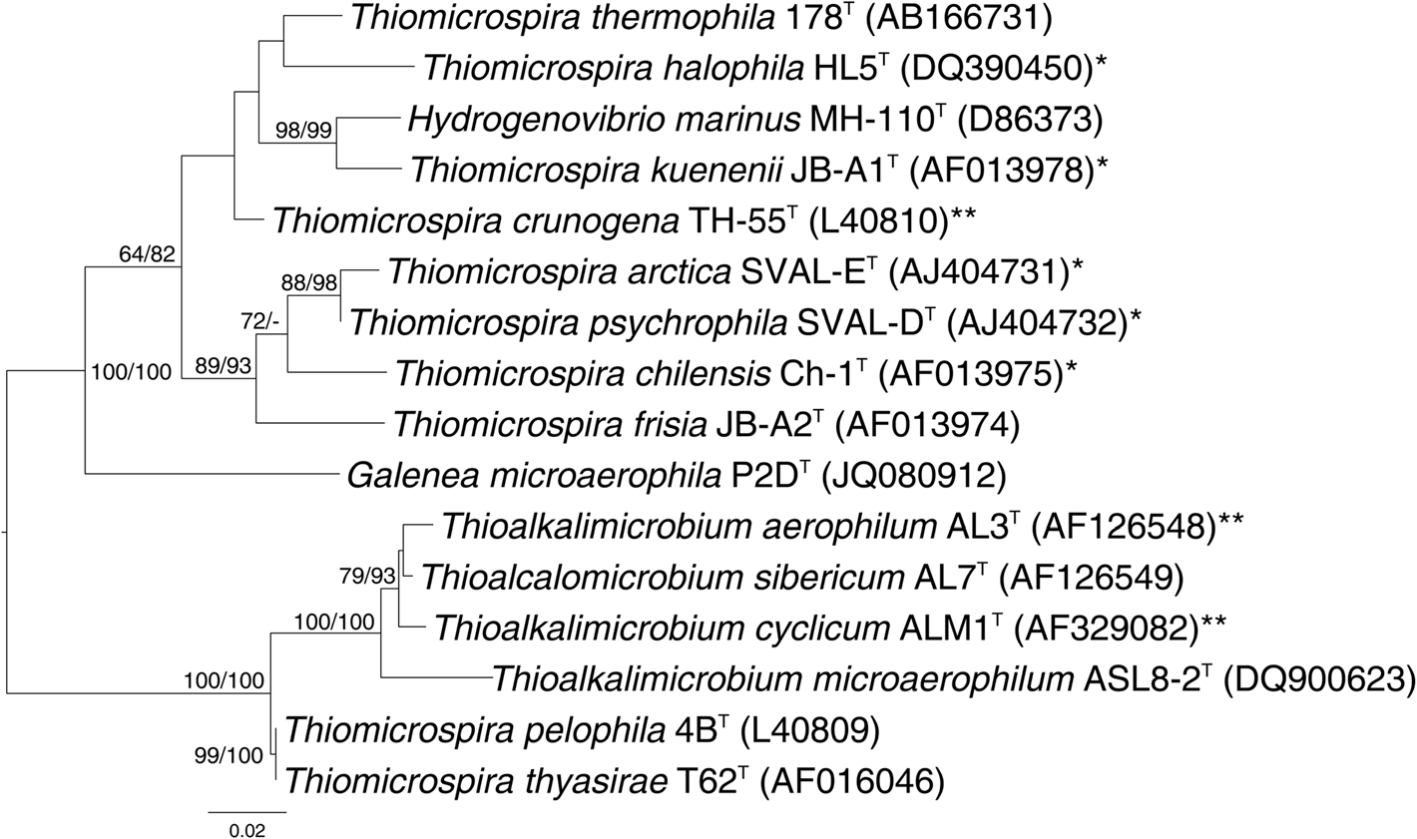
Phylogenetic tree highlighting the position of *T. cyclicum* within the family *Piscirickettsiaceae* *. T. cyclicum* is shown relative to the type strains of *Thioalkalimicrobium* and all species from the three closest related genera (*Thiomicrospira*, *Hydrogenovibrio* and *Galenea*) within the family. The tree was inferred from 1,530 aligned characters [58, 59] of the 16S rRNA gene sequence under the ML criterion [60]. The rooting shown was inferred by the midpoint-rooting method [36]. The branches are scaled in terms of the expected number of substitutions per site (bar). Numbers adjacent to the branches are support values from 1,000 ML bootstrap replicates [61] (left) and from 1,000 maximum-parsimony bootstrap replicates [62] (right) if larger than 60 %. Lineages with type strain genome sequencing projects registered in GOLD [7] are labeled with one asterisk, those also listed as ‘Complete and Published’ with two asterisks (see [37] and AGFA00000000 for *T. aerophilum*)

Figure 1 shows the phylogenetic neighborhood of *T. cyclicum* in a 16S rRNA based tree. The sequences of the two identical 16S rRNA gene copies in the genome differ by one nucleotide and a nine bp long gap from the previously published 16S rRNA sequence (AF329082), which contained nine ambiguous base calls.

The paraphyletic structure of the genus *Thiomicrospira* in Fig. 1 and the location of *Hydrogenovibrio marinus* and *Galenea microaerophila* within *Thiomicrospira* might indicate the need for genome sequence-based reclassifications once enough reference sequences become available.

**Fig. 2:**
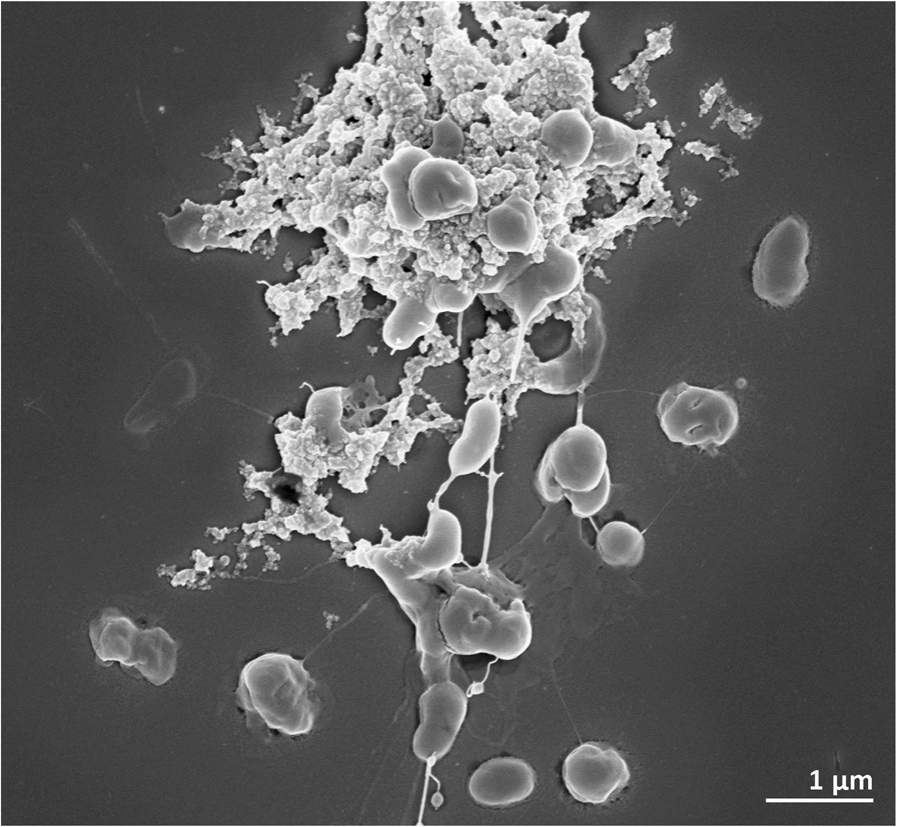
Electron micrograph of *T. cyclicum* ALM1 DSM 14477^T^. Micrographs of bacterial cells grown from a culture of DSM 14477^T^ in DSMZ medium 925 at 28 °C were taken with a field-emission scanning electron microscope (FE-Merlin, Zeiss). Bacteria were fixed with formaldehyde and glutaraldehyde, washed with TRIS-EDTA buffer, dehydrated with a graded series of acetone, critical-point dried with liquid CO_2_, sputter coated with gold-palladium and imaged in a Zeiss Merlin with the HE-SE2 detector and inlens-detector in a 25:75 ratio with an acceleration voltage of 5 kV

Cells of *T. cyclicum* ALM 1^T^ are non-motile, Gram-negative staining, irregular spheres often in the form of open rings with a diameter of 0.5–0.8 μm and a cell width of 0.3–0.4 μm (Table 1 and Fig. 2) [1]. Carboxysome-like structures were frequently observed (see [1]). Colonies of strain ALM 1^T^ are reddish, transparent with a diameter up to 3 mm [1]. Cells oxidize thiosulfate and sulfide but grow less actively on polysulfide and tetrathionate [1]. The pH range for growth is 6.5 to 11 (optimum 9.5) with a moderate salt concentration (about 0.6 M NaCl) [1].

**Table 1:** Classification and general features of *T. cyclicum* ALM1^T^ in accordance with the MIGS recommendations [48] (published by the Genome Standards Consortium [49]) and the NamesforLife database [50]

MIGS ID	Property	Term	Evidence code^a^
	Classification	Domain *Bacteria*	TAS [51]
		Phylum *‘Proteobacteria’*	TAS [52]
		Class *Gammaproteobacteria*	TAS [53, 54]
		Order *Thiotrichales*	TAS [54, 55]
		Family *Piscirickettsiaceae*	TAS [54, 56]
		Genus *Thioalkalimicrobium*	TAS [2]
		Species *Thioalkalimicrobium cyclicum*	TAS [1]
		(Type) strain: ATM^T^ (AF329082)	
	Gram stain	negative	TAS [1, 2]
	Cell shape	open ring-shaped	TAS [1]
	Motility	non-motile	TAS [1]
	Sporulation	not reported	
	Temperature range	mesophile, about 28 °C	NAS
	Optimum temperature	not reported	
	pH range; Optimum	not reported	
	Carbon source	CO_2_	NAS
MIGS-6	Habitat	water	TAS [1]
MIGS-6.3	Salinity	Moderate, 0.6 to 1.5 M NaCl	TAS [1]
MIGS-22	Oxygen requirement	aerobic	TAS [1]
MIGS-15	Biotic relationship	free-living	TAS [1]
MIGS-14	Pathogenicity	none	NAS
MIGS-4	Geographic location	Lake Mono (California)	TAS [1]
MIGS-5	Sample collection	1999	TAS [1]
MIGS-4.1	Latitude	38.012	TAS [1]
MIGS-4.2	Longitude	−118.976	TAS [1]
MIGS-4.4	Altitude	1926 m	TAS [1]

^a^Evidence codes - *IDA* Inferred from Direct Assay, *TAS* Traceable Author Statement (i.e., a direct report exists in the literature), *NAS* Non-traceable Author Statement (i.e., not directly observed for the living, isolated sample, but based on a generally accepted property for the species, or anecdotal evidence). These evidence codes are from the Gene Ontology project [57]

### Chemotaxonomic data

The original description of strain ALM 1^T^ [1] did not provide any chemotaxonomic information. No new chemotaxonomical data were generated for this report.

## Genome sequencing information

### Genome project history

This organism was selected for sequencing as part of the DOE JGI CSP 2008. The genome project is deposited in the Genomes On Line Database [7] and the complete genome sequence is deposited in GenBank. Sequencing, finishing and annotation were performed by the DOE JGI using state of the art sequencing technology [8]. A summary of the project information is shown in Table 2.

**Table 2:** Project information

MIGS ID	Property	Term
MIGS 31	Finishing quality	Finished
MIGS-28	Libraries used	One 454 pyrosequence standard library, one 454 PE library (9 kb insert size), one Illumina library
MIGS 29	Sequencing platforms	Illumina GAii, 454 GS FLX Titanium
MIGS 31.2	Fold coverage	1,521.2 × Illumina, 38.2 × pyrosequence
MIGS 30	Assemblers	Newbler version 2.3, Velvet version 0.7.63, phrap version SPS – 4.24
MIGS 32	Gene calling method	Prodigal
	Locus Tag	Thicy
	Genbank ID	CP002776
	GenBank Date of Release	July 6, 2012
	GOLD ID	Gc01777
	BIOPROJECT	PRJNA52629
MIGS 13	Source Material Identifier	DSM 14477
	Project relevance	Biotechnological, Bioremediation

### Growth conditions and genomic DNA preparation

Strain ALM 1^T^ was grown from a culture of DSM 14477^T^ in DSMZ medium 925 at 28 °C. gDNA was purified using the Genomic-tip 100 System (Qiagen) following the directions provided by the supplier. The purity, quality and size of the bulk gDNA preparation were assessed by JGI according to DOE-JGI guidelines which included electrophoretic separation of samples and comparison against standards of known molecular masses, analysis of UV absorption spectra and sequencing of the 16S rDNA.

### Genome sequencing and assembly

The genome was sequenced using a combination of Illumina and 454 sequencing platforms. All general aspects of library construction and sequencing can be found at the JGI website [64]. Pyrosequencing reads were assembled using the Newbler assembler [9]. The initial Newbler assembly consisted of 15 contigs in one scaffold and the consensus contigs were computationally shredded to form 2 kb overlapping reads. Illumina GAii sequencing data (3,091 Mb) were assembled with Velvet [10] and the consensus sequences were computationally shredded into 1.5 kb overlapping reads. The computational shreds from both assemblies were assembled together with the 454 long-insert paired end reads using phrap [11, 12]. The 454 draft assembly was based on 171.4 Mb 454 draft data and all of the 454 paired end data. The Phred/Phrap/Consed software package [11–13] was used for sequence assembly and quality assessment in the subsequent finishing process. After the shotgun stage, reads were assembled with parallel phrap (High Performance Software, LLC). Possible mis-assemblies were corrected with gapResolution [14, 65], Dupfinisher [15], or sequencing cloned bridging PCR fragments with subcloning. Gaps between contigs were closed by editing in Consed, by PCR and by bubble PCR primer walks [16] (J.-F. Chang, unpublished). A total of 74 additional reactions and one shatter library were necessary to close gaps and to raise the quality of the final sequence. Illumina reads were also used to correct potential base errors and increase consensus quality using a software Polisher developed at JGI [17]. The error rate of the final genome sequence is less than 1 in 100,000. Together, the combination of the Illumina and 454 sequencing platforms provided 1559.4 × coverage of the genome. The final assembly contained 216,642 pyrosequence and 38,029,488 Illumina reads.

### Genome annotation

Genes were identified using Prodigal [18] as part of the DOE-JGI [8] genome annotation pipeline, followed by a round of manual curation using the JGI GenePRIMP pipeline [19]. The predicted CDSs were translated and used to search the NCBI non-redundant database, UniProt [20], TIGRFam [21], Pfam [22], PRIAM [23], KEGG [24], COG [25], and InterPro [26] databases. These data sources were combined to assert a product description for each predicted protein. Additional gene prediction analysis and functional annotation was performed within the IMG-ER platform [27].

## Genome properties

**Fig. 3:**
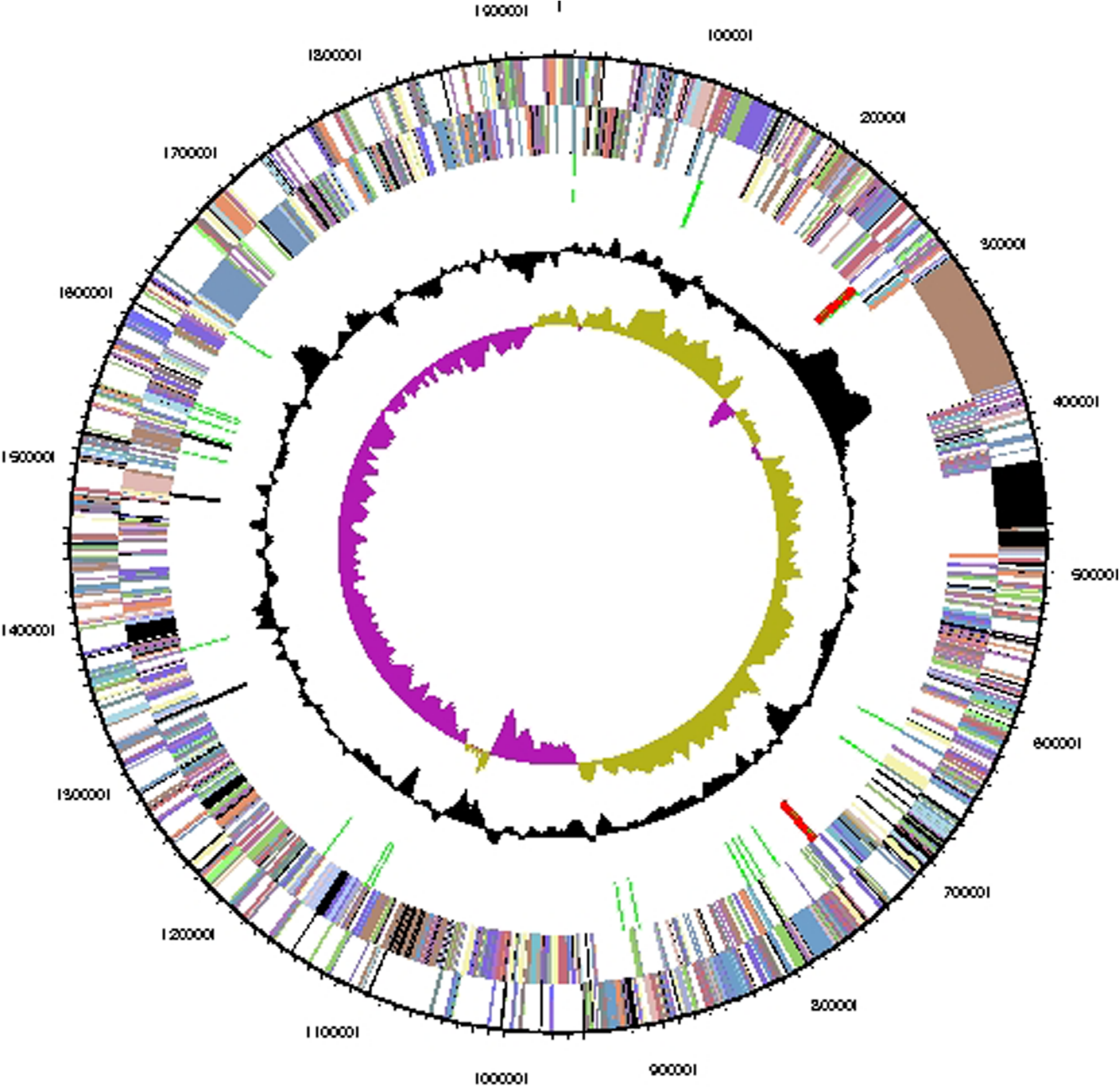
Graphical map of the chromosome. From outside to the center: Genes on forward strand (color by COG categories), Genes on reverse strand (color by COG categories), RNA genes (tRNAs green, rRNAs red, other RNAs black), GC content (black), GC skew (purple/olive)

The genome consists of a circular 1,932,455 bp chromosome with 47 % G + C content (Table 3 and Fig. 3). Of the 1734 genes predicted, 1684 were protein-coding genes, and 50 RNAs; 19 pseudogenes were also identified. The majority of the protein-coding genes (78.5 %) were assigned a putative function while the remaining ones were annotated as hypothetical proteins. The distribution of genes into COGs functional categories is presented in Table 4.

**Table 3:** Genome statistics

Attribute	Value	% of Total
Genome size (bp)	1,932,455	100.0
DNA coding (bp)	1,818,441	94.1
DNA G + C (bp)	907,872	47.0
DNA scaffolds	1	
Total genes	1,734	100.0
Protein coding genes	1,684	97.1
RNA genes	50	2.9
Pseudo genes	19	1.1
Genes in internal clusters	543	31.3
Genes with function prediction	1,361	78.5
Genes assigned to COGs	1,459	84.1
Genes with Pfam domains	1,502	86.6
Genes with signal peptides	124	7.2
Genes with transmembrane helices	357	20.6
CRISPR repeats	0

**Table 4:** Number of genes associated with general COG functional categories

Code	Value	%age	Description
J	153	9.7	Translation, ribosomal structure and biogenesis
A	1	0.1	RNA processing and modification
K	71	4.5	Transcription
L	86	5.4	Replication, recombination and repair
B	0	0.0	Chromatin structure and dynamics
D	27	1.7	Cell cycle control, Cell division, chromosome partitioning
V	28	1.8	Defense mechanisms
T	48	3.0	Signal transduction mechanisms
M	117	7.4	Cell wall/membrane biogenesis
N	12	0.8	Cell motility
U	56	3.5	Intracellular trafficking and secretion
O	88	5.6	Posttranslational modification, protein turnover, chaperones
C	95	6.0	Energy production and conversion
G	59	3.7	Carbohydrate transport and metabolism
E	123	7.8	Amino acid transport and metabolism
F	49	3.1	Nucleotide transport and metabolism
H	109	6.9	Coenzyme transport and metabolism
I	40	2.5	Lipid transport and metabolism
P	89	5.6	Inorganic ion transport and metabolism
Q	26	1.6	Secondary metabolites biosynthesis, transport and catabolism
R	170	10.8	General function prediction only
S	135	8.5	Function unknown
-	275	15.9	Not in COGs

The total is based on the total number of protein coding genes in the genome

## Insights from the genome sequence

*T. cyclicum* has been described as an obligate chemolithoautotroph, and its genome contains only 1684 protein encoding genes indicating a reduction in gene contents possibly in adaptation to this lifestyle. Reductions in genome size are a common feature in bacteria from many specialized ecological niches where relatively stable growth conditions are encountered. Examples include other free-living bacteria in the family *Piscirickettsiaceae*, *Thioalkalimicrobium* species*,**Thiomicrospira crunogena* and related species, although in most cases the genome reduction is not as extreme as in this case and ~ 2000 proteins are present.

**Fig. 4:**
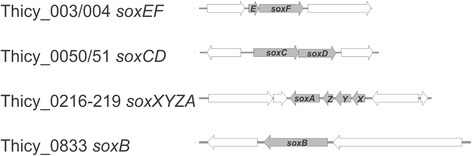
Schematic representation of the gene regions encoding enzymatic components of the Sox (**S**ulfur **Ox**idation) pathway; *sox* genes are shown in grey, all genes encoding proteins not related to the Sox complex are showing in white. Gene numbers and genes encoded are shown in the figure

During chemolithoautotrophic growth, strain ALM 1^T^ oxidizes reduced sulfur compounds such as thiosulfate or sulfide without the formation of sulfur as an intermediate, which suggests that it uses a Sox-type sulfur oxidation pathway [28] rather than a combination of DSR and Sox proteins which leads to the formation of elemental sulfur as an intermediate and seems to be common in other *Gammaproteobacteria*, such as the phototrophic purple sulfur bacteria [29]. The Sox sulfur oxidation pathway relies on four essential enzyme complexes, SoxAX, SoxYZ, SoxB, and SoxCD, to oxidize reduced sulfur compounds to sulfate without the formation of free intermediates [28, 30, 31], and all of these proteins are encoded in the *T. cyclicum* genome. In the reaction cycle of the Sox multienzyme complex the SoxAX cytochrome (encoded by Thicy_216 & Thicy_219) that catalyzes covalent attachment of reduced sulfur compounds such as thiosulfate to the SoxYZ carrier protein (Thicy_217, Thicy_218), the manganese-containing SoxB (Thicy_0833) protein then removes the fully oxidized sulfur residues from SoxYZ through hydrolysis, while the SoxCD sulfane dehydrogenase (Thicy_50, Thicy_51) a heterotetrameric complex of the molybdenum protein and a cytochrome, catalyzes a six electron oxidation of reduced sulfur residues bound to SoxYZ [32–36] (Fig. 4). All *T. cyclicum* Sox proteins are most closely related to homologues found in *Thioalkalimicrobium aerophilum* (94–95 % amino acid identity) as well as *Thiomicrospira sp.* and *Hydrogenovibrio marinus*, which is in keeping with the phylogenetic position of this bacterium.

In *T. cyclicum* the *sox* genes encoding the essential components of the Sox multienzyme complex are distributed in three separate genomic gene loci (Fig. 4). This is similar to what has been seen in the related *Thiomicrospira* species [37], but differs from the situation in other *Proteobacteria*, e.g. the α-Proteobacterium *Paracoccus pantotrophus*, where the genes encoding the core Sox enzymes as well as genes encoding accessory Sox proteins such as SoxV, W, H, S or the SoxF sulfide dehydrogenase are located in the same gene region and often even within only one or two major operons [28, 38].

In *T. cyclicum* ALM 1, most of genes encoding accessory Sox proteins appear to be absent, only a homologue of the SoxF flavocytochrome (Thicy_0003, Thicy_0004) (Fig. 4) and a gene encoding a homologue of the SoxH protein (Thicy_0412), the exact function of which is unknown, were detected during our analyses of the genome. This leads to the question of how essential these accessory proteins are for the function of the Sox complex. *T. cyclicum*, unlike many of the sulfur oxidizing α-*Proteobacteria*, is an obligate chemolithoautotroph and thus relies on the optimal function of this pathway for energy generation, and yet does not appear to rely on the accessory proteins to keep the core Sox enzymes functional.

**Fig. 5:**
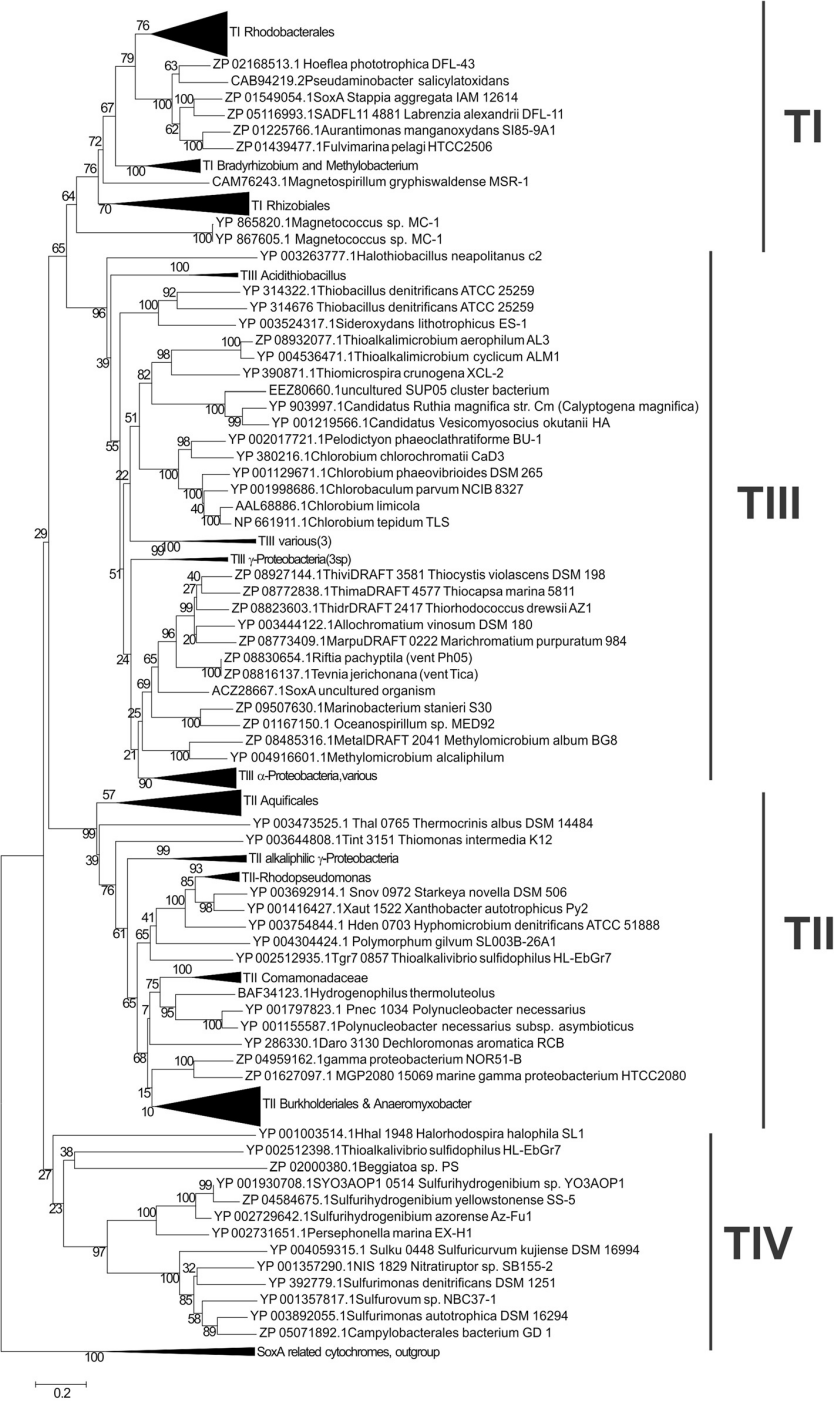
Phylogenetic analysis of SoxA cytochromes. The phylogenetic tree was generated using the Neighbor-joining algorithm as integrated into the Mega program suite [63], bootstrap values are based on 500 replicates

Studies of the SoxAX cytochromes in various *Proteobacteria* have led to the realization that these proteins are extremely diverse. There are currently three recognized types of proteins that vary significantly in terms of the redox cofactors present as well as their subunit structure and specifically the sequences of the SoxX proteins involved in the SoxAX complexes [30, 31]. In comparison to biochemically characterized SoxA proteins, the protein encoded by the *T. cyclicum**soxA* gene is most closely related to the Type III SoxA protein from *Allochromatium vinosum* (33.9 % amino acid sequence identity, as opposed to 21.8 and 17.4 % identity with the Type I and Type II SoxA proteins from *P. pantotrophus* and *S. novella*) (Fig. 5). Type III SoxAX proteins normally contain three subunits, SoxA, SoxX and SoxK [39]. The low molecular weight SoxK protein is required to stabilize the complex of SoxA and SoxX. In *T. cyclicum*, however, no gene encoding a protein homologous to SoxK appears to be present, which indicates that there is even more diversity of SoxAX proteins than previously assumed. A similar situation was already described by Ogawa et al. [39] for the SoxAX protein from the related bacterium, *Thiomicrospira crunogena*, and has also been discussed in depth, including a phylogenetic analysis across all groups of SoxA related proteins in two recent reviews [30, 31] (Fig. 5).

Although the Sox sulfur oxidation pathway has been recognized as a key pathway in microbial sulfur chemolithotrophy, issues still exist with the annotation of the various genes in automated annotation pipelines. For example, *soxB* genes are often annotated as encoding a ‘5′nucleotidase’, which is correct as SoxB does belong to this larger group of enzymes, but at the same time creates confusion as to the actual nature of the encoded protein. Dedicated SoxB protein domains exist (e.g. cd07411, abbreviated as “MPP_soxB_N” or SoxB proteins with an N-terminal metallophosphatase domain), and recently a dedicated full length domain, TIGR04486 (thiosulf_soxB) has been defined.

Another curiosity is the annotation of the SoxCD sulfane dehydrogenase (Thicy_0050/0051) as a ‘SO (sulfite oxidase) family protein’ (which is correct), and then as a ‘nitrate reductase (NADH)’. In as far as is currently known, the sulfite oxidase enzyme family only contains nitrate reductases from plants, and no prokaryotic nitrate reductases have ever been found in this enzyme family. Clearly, there is scope for improving the specificity of current COGs/cd patterns to avoid such obvious errors in the future, although the conserved domain cd_02113 is diagnostic for SoxC proteins, regardless of their annotation.

We also analyzed the *T. cyclicum* genome for other proteins known to be involved in sulfur oxidation that are not part of the Sox multienzyme complex. Such proteins are frequently found in sulfur oxidizing bacteria and enhance their ability to use different sulfur compounds, including those that are not generally recognized as substrates of the Sox pathways (e.g. tetrathionate) or to cope with toxic sulfur compounds that can be byproducts of abiotic sulfur conversions (e.g. sulfite and sulfide converting enzymes). No genes encoding homologues of DSR, APS reductase (*aprABM*), tetrathionate hydrolase (*tth* gene) or sulfite dehdyrogenases (*sorAB*) were identified. However, we did identify two genes (Thicy_0064 & Thicy_1132) that encode proteins with strong similarities to proteins annotated as SQRs in *T. crunogena* (Tcr_1170, Tcr_1381) [37].

The protein encoded by the putative SQR gene Thicy_1132 is actually related to Ndh NADH dehydrogenase-type proteins, while Thicy_0064 shows homology to ‘HcaD uncharacterized FAD dependent dehydrogenases, COG0446′. Using the SQR classification system of [40], the two *T. cyclicum* SQRs could be classified as a periplasmic (34 aa Tat- leader peptide; 52 % conserved aa) SqrB type protein (Thicy_0064), and a soluble, likely cytoplasmic SqrF-like protein (Thicy_1132, 49 % conserved aa). Interestingly, the Thicy_1132 encoded protein only has homology to the SqrF like proteins, while the Thicy_0064 encoded protein exhibited significant homologies to all SQR types except SqrE and SqrF. It would thus appear that *T. cyclicum* contains two SQRs of different types, as representatives from both SqrB and SqrF groups have been enzymatically characterized.

Overall despite the fact that sulfur oxidation is a key element of *T. cyclicum* metabolism, the actual number of genes supporting this process is very small and shows very little redundancy or diversity. All genes encoding essential proteins of the Sox pathway are present as single copies, and genes encoding other enzymes known to support chemolithotrophic growth on sulfur compounds are absent. This is in contrast to other sulfur oxidizing bacteria such as the haloalkaliphilic *Thioalkalivibrio sulfidophilus* [41] which contains several copies of genes encoding SoxAX proteins and *Starkeya novella* where two copies of SoxAX and SoxYZ encoding genes are present [42], as well as additional genes encoding sulfur converting enzymes that are not part of the Sox complex.

With chemolithoautotrophy being the major growth mode for *T. cyclicum*, we also investigated the carbon dioxide fixation pathways present in this bacterium. Of the known microbial pathways for carbon dioxide fixation only the Calvin Benson Bassham cycle was present, and carbon dioxide incorporation into phosphoenolpyruvate to form oxaloacetate, a required intermediate of the TCA cycle, was also identified using the KEGG pathway database [24].

Central carbon metabolism in *T. cyclicum* includes a complete set of genes encoding glycolysis and the pentose phosphate pathway as well as a pyruvate dehydrogenase enzyme complex and several routes by which pyruvate can be converted into oxaloacetate (PEP synthase, Thicy_1283, EC 2.7.9.2; PEP carboxylase, Thicy_1240, EC 4.1.1.31) or lactate (D-lactate dehydrogenase, Thicy_1457, 1.1.1.28)

The TCA cycle of *T. cyclicum* is incomplete, with genes encoding the 2-oxoglutarate dehydrogenase or homologous enzymes (e.g. 2-oxoglutarate:ferredoxin oxidoreductase, KorAB) not having been identified in the genome. This indicates that in *T. cyclicum* the TCA cycle mainly serves biosynthetic purposes rather than being part of general energy generation, which is in keeping with the chemolithoautotrophic lifestyle of this bacterium, as sulfur oxidation by the Sox pathway or via SQRs will feed electron directly into the respiratory chain for energy generation.

The respiratory chain of *T. cyclicum* is of a very linear architecture, with only complex I being represented by three different types of NADH dehydrogenases. A multisubunit (‘mitochondrial type’) NADH dehydrogenase (EC 1.6.5.3) is encoded by the *nuo* gene cluster (Thicy_0637–0650), while the other two are encoded by two genes (EC 1.6.99.1, Thicy_1224–1225) and a single gene (EC 1.6.99.3, Thicy_0083), respectively. Complex II/succinate dehydrogenase is encoded by genes Thicy_0875–0878, while Complex III/cytochrome bc1-complex is encoded by Thicy_0482–484. Only a single gene cluster encoding a cytochrome *c* oxidase appears to be present (Thicy_1535–1529), which encodes a *cbb*_*3*_-type cytochrome oxidase. This type of cytochrome oxidase is known to have a high affinity for oxygen and thus has been associated with microaerophilic growth conditions [43, 44], suggesting that in its natural environment *T. cyclicum* encounters medium to low oxygen tensions. In addition to this function, *cbb*_*3*_-type cytochrome oxidases have been implicated in affecting various regulatory processes in bacterial cells [45–47], including redox regulation and responses to environmental conditions, and it is possible that the enzyme from *T. cyclicum* also fulfills additional, regulatory functions. An F-type ATPase (Thicy_1606–1612) completes the respiratory chain.

## Conclusions

With only about ~ 1700 encoded genes the genome of *T. cyclicum* ALM1^T^ is relatively small compared to genomes from related sulfur oxidizing bacteria such as *Thiomicrospira sp.* or *Thioalkalivibrio sp.* which generally contain ~2000 or more protein encoding genes. The reduction in genome size becomes even more obvious in comparison to other sulfur chemolithoautotrophic bacteria (e.g. *Starkeya novella*, *Paracoccus* sp., *Rhizobiales**sp.*) that often have more than 4000 encoded genes and also tend to encode redundant pathways. This again is likely to reflect the limited availability of substrates for energy generation in the organism’s natural habitat, which is an extreme environment with high alkalinity and salinity.

Despite the reliance of *T. cyclicum* on autotrophy for acquiring cell carbon, only a single pathway for carbon dioxide fixation was found, and only the Sox pathway for sulfur oxidation and a few additional proteins that enable efficient use of sulfide as an energy source (SQRs and flavocytochromes) were identified. This is in keeping with a direct oxidation of sulfur substrate such as thiosulfate and sulfide to sulfate without intermediate formation of elemental sulfur which is a trait of the other major sulfur oxidation pathway that uses the DsrAB dissimilatory sulfite reductase. It is also supported by our observation on aerobic cultures of *T. cyclicum* supplement with thiosulfate as an energy source, which showed no sign of sulfur formation, which would have led to increased, optically apparent, turbidity of the culture during growth.

However, with about 20 % of genes having either unknown functions or not being assigned a COG category, there are clearly many things that can still be discovered regarding this organism. The apparent absence of accessory genes aiding in the maturation of the essential Sox sulfur oxidation enzymes is unusual, and should be further investigated, as should the effect of a high pH environment on the physical and catalytic properties of the periplasmic Sox proteins. *T. cyclicum* also prefers moderate salt concentrations, and it would be interesting to carry out comparative studies on compatible solutes and other adaptations between species of haloalkaliphilic sulfur oxidizers.

## Taxonomic and nomenclatural proposals

The difference in the reported G + C content of *T. cyclicum* (49.6 %) [1] to the one calculated from the genome sequence (47.0 %) calls for an emendation of the species description. The genome sequence-derived G + C content is also outside of the 48 to 51.2 % G + C range reported for the genus *Thioalkalimicrobium* [2].

## Emended description of the species *Thioalkalimicrobium cyclicum* Sorokin et al. 2002

The description of the species *Thioalkalimicrobium cyclicum* is the one given by Sorokin *et al.* 2002 [1], with the following modification. The G + C content, rounded to zero decimal places, is 47 %.

## Abbreviations

CSP: Community Sequencing Program
DSM: Deutsche Sammlung von Mikroorganismen
DSMZ: Deutsche Sammlung von Mikroorganismen und Zellkulturen (German Collection of Microorganisms and Cell Cultures
DSR: dissimilatory sulfite reductase
HSP: high-scoring segment pair
IMG-ER: integrated microbial genomes - expert review
IUMS: international union of microbiological societies
JCM: Japan collection of microorganisms
ML: maximum likelihood
PEP: phosphoenolpyruvate
SOX: sulfur oxidation
SQR: Sulfide: quinone reductase
